# Perceptions of Covid-19 lockdowns and related public health measures in Austria: a longitudinal online survey

**DOI:** 10.1186/s12889-021-11476-3

**Published:** 2021-08-04

**Authors:** Agata Łaszewska, Timea Helter, Judit Simon

**Affiliations:** Medical University of Vienna, Center for Public Health, Department of Health Economics, Kinderspitalgasse 15, 1090 Vienna, Austria

## Abstract

**Background:**

Introducing national lockdown has been effective in containing Covid-19. However, several studies indicated negative impacts of lockdowns on the well-being and mental health of many people. In Austria, the first Covid-19-related lockdown was introduced on 16 March 2020 with most restrictions being lifted 1 month later. Seven months after that, in November 2020, the second full lockdown was implemented. The aim of this study was to compare the perceptions and experiences of the general population related to the first and second Covid-19 lockdowns in Austria.

**Methods:**

Two waves of an online survey were conducted in May and December 2020 asking respondents about their concerns related to the Covid-19 illness, personal experiences of the lockdowns, perceptions of and compliance with imposed public health measures, and the impact of the Covid-19 pandemic on different aspects of life during the two lockdowns. Descriptive statistics including frequency analysis were used to compare respondents’ answers collected in the two waves of the survey. T-test and chi-square tests were used to test differences between the two lockdowns.

**Results:**

Five hundred sixty participants were included in the first wave and a sub-sample of 134 participants in the second wave of data collection. During the second lockdown, study respondents were more concerned about their family members contracting Covid-19 when compared with the first lockdown. Compliance with public health measures was overall lower during the second lockdown, although it varied according to the type of the measure. Closure of schools was seen as the least essential restriction during the second lockdown, while wearing masks gained additional approval between the first and the second lockdown. Larger negative impacts of the Covid-19 pandemic on friendships, leisure activities, education and community were reported during the second lockdown.

**Conclusions:**

The study found that the extended duration of the pandemic and recurring lockdowns restricting freedom of movement and social contacts appear to have caused significant disruptions to many areas of life. Furthermore, declining adherence to most public health measures over time raises a question about the effectiveness of future lockdown measures.

**Supplementary Information:**

The online version contains supplementary material available at 10.1186/s12889-021-11476-3.

## Introduction

Lockdowns during the Coronavirus disease 2019 (Covid-19) outbreak include a range of largely non-pharmaceutical interventions to limit physical interactions and introduce social distancing [1]. Although the introduction of a lockdown is an effective strategy to slow the spread of infection [2], several studies pointed out its negative effects on mental health [3–5], health behaviours [6], social connectedness, and loneliness [4]. A recent report presented by the Association of Schools of Public Health in the European Region found that lockdown restrictions contributed to an increase in problems such as addiction, poor diet, lack of physical activity, deteriorating mental health, and domestic violence [7]. As these lockdowns have so negatively affected individuals in these ways and have had a devastating impact on economic growth, public debt and employment [8], lockdown measures should be administered carefully and should be based on unbiased carefully collected data [9, 10]. The adverse effects of the lockdowns should not be ignored and officials should take every measure to minimise the societal impact and ensure that this experience is as tolerable as possible for the general population [11–14]. Therefore, the evaluation of lockdown strategies and containment policies is crucial. In a cross-sectional study, Sabat et al. found that citizens of seven European countries (Denmark, France, Germany, Italy, Portugal, the Netherlands, and the UK) were generally satisfied with their government’s responses to the Covid-19 outbreak [15]. To the best of our knowledge, there are only a few studies published so far that assess policy responses and people’s perceptions of the implemented public health measures during the Covid-19 outbreak and their compliance with the government-advised preventive measures across several time-points.

### Lockdowns in Austria

Austria initially reacted quickly to the Covid-19 pandemic and was praised for the policies it implemented to contain the virus [16]. The first lockdown was introduced in March and the second one in November. Both required all non-essential business and schools to close and reduce social contacts. According to the Oxford Covid-19 government response stringency index, at the time of the first and the second lockdown, the score for Austria on a scale from 0 to 100 (100 = strictest) was 81.48 and 82.41, respectively [17].

The first case of Covid-19 in Austria was diagnosed on 25 February 2020 [18]. The first lockdown was imposed 20 days later on 16 March when the cases grew to 1192 [19]. Stores selling non-essential goods as well as bars, restaurants, federal parks, sports facilities and public baths were forced to close. Supermarkets, chemist’s shops and pharmacies remained open. Air traffic was largely suspended. Strict contact regulations and curfews based on the Covid-19 law came into force [20]. On 26 March a peak of new daily cases (1050 cases) was reported and a peak of daily deaths from Covid-19 followed on 6 April with 31 deaths reported [19]. The number of active cases started to decrease after reaching its peak (8981 active cases) in the beginning of April 2020, which led to an easing of restrictions beginning 14 April.

After a summer with fairly low case numbers, a steeper growth curve was observed from September onwards with a dramatic increase in new daily cases in October [16, 19]. While at the end of September 8602 active Covid-19 cases were reported in Austria, this number grew to 43,187 by 31 October [19], an increase of 400%. On 11 November 2020 a peak of new daily infections was reported at 9216 confirmed cases.

Based on the success of the first lockdown in reducing the spread of the virus, the Austrian government implemented a second lockdown to hinder the rapidly growing pandemic curve. On 3 November a “light” lockdown was imposed which quickly turned into another “hard” national lockdown that began on 17 November. The restrictions included closure of cafes, restaurants, hairdressers and beauty salons, and all shops except those providing essential services and selling essential goods (e.g. grocery stores, pharmacies, post offices, gas stations, etc.) with an obligatory closing time of 7:00 pm. Primary schools joined secondary schools and universities in moving to distance learning. A 24-h curfew was introduced and people were allowed to leave their homes only for essential purposes such as: caring for other people or animals, family duties, outdoor exercise, and visiting religious institutions. Visits in hospitals and nursing homes were reduced to one visit per week. In public spaces, one meter distance between persons outside the same households was required as well as compulsory masks in indoor spaces and public transport. Only take-away and delivery was allowed for restaurants, and hotels and accommodation establishments closed, with an exception for business trips. Events were prohibited, and sports and leisure facilities were closed. Home office was recommended when possible but was not mandatory [21]. This lockdown remained in effect until 6 December.

### Aim of the study

Studies conducted in Austria reported many negative impacts of the Covid-19 lockdowns including increased loneliness [22], worsening of mental health [23–26], decreased quality of life [24], and decreased engagement in sports [27]. In terms of the long-term impacts, one study reported that depression did not improve in the weeks after lifting lockdown measures [28]. The Austrian Corona Panel Project showed further negative effects of the Covid-19 lockdowns [29] in terms of the economic, political and health aspects such as loss of income especially among low income households, loss of trust in the parliament and the federal government, and an increase in the cigarette and alcohol consumption during the first lockdown [30–33]. Due to the negative consequences of the lockdowns and of the pandemic, nuanced evaluation of policies is crucial for the design of future restrictions implemented to contain the spread of Covid-19. In this study, the aim was to assess and compare the general population’s experiences of the Covid-19 situation and their attitudes towards public health measures during the two lockdowns in spring and fall of 2020 in Austria.

## Methods

### Recruitment of study participants

Study participants were recruited using convenience sampling, i.e. people who self-selected to become part of a study when responding to an advert. Any adult over 18 years with sufficient German knowledge and main residency in Austria was able to participate.

The first wave of the survey was conducted between 27 May and 16 June 2020, with all questions referring to the one-month lockdown period in Austria between 16 March and 15 April 2020. The weblink for the online survey was distributed via social media platforms (Facebook and Twitter) and by directly contacting several institutions across Austria (e.g. universities, sport clubs, the Red Cross, non-profit mental health organisations such as pro mente). Information about the study was shared on the Facebook and Twitter sites of the Medical University of Vienna and through various Facebook groups related to Covid-19 (e.g. Coronavirus Österreich, Coronavirus Österreich Info, Das Coronavirus (Covid-19) Hilfe & Erfahrungen & Austausch). To reach a more diverse study sample, information about the study was posted to the comments sections under Covid-19-related articles on Facebook that were shared by the most popular newspapers in Austria (e.g. Der Standard, Die Presse, Ö24, Heute and local online newspapers). As part of the first wave of data collection, all recruited participants were invited to provide their e-mail addresses at the end of the survey and agree to be contacted for follow-up data collection.

The second wave of the study was conducted between 2 December and 9 December 2020 with questions referring to the second national lockdown in Austria between 17 November and 6 December 2020. Those participants, who provided their e-mail addresses in the first round of data collection, were contacted and a link to the second survey was provided.

### Survey design

The survey was developed in the SoSci online survey platform [34]. The survey was conducted in German and consisted of questions about socio-demographics, Covid-19-related questions (including information about Covid-19 infections), lockdown-related questions (including the perceptions of the public health measures in place during the lockdown in Austria). The questionnaire used in the study (translated to English) is provided in Supplementary file 1. Further details of the study design and recruitment can be found in Simon et al. [26].

### Data analysis

Collected data were checked for inconsistencies. Any entry with time of completion below 7 min was deleted from the dataset. Data were analysed primarily using descriptive statistics. Mean values and standard deviation were reported for continuous variables and frequencies were reported for categorical variables.

Data related to the personal experience of the Covid-19 lockdowns were collected on a five-point Likert scale (1 – Strongly disagree, 2 – Slightly disagree, 3 – Neutral, 4 – Slightly agree, 5 – Strongly agree) with higher scores presenting higher level of agreement with the statement presented to the study participants. Data were summarised using means and standard deviations for ease of comparison of the answers between the two lockdowns. Differences between the two lockdowns were assessed using the chi-square test for categorical variables.

Variables related to the perceptions of the necessity of imposed public health measures and compliance with the public health measures were collected on a scale from 1 to 10 (ranging from 1 ‘Completely unnecessary’ to 10 ‘Absolutely essential’; and from 1 ‘Not complying at all’ to 10 ‘Complying completely’). These data were re-coded to a 5-point scale: 1 – Completely unnecessary (answers 1, 2), 2 – Unnecessary (answers 3, 4), 3 – Neutral (answers 5, 6), 4 – Necessary (answers 7, 8), 5 – Absolutely essential (answers 9, 10); and 1 – Not complying at all (answers 1, 2), 2 – Not always complying (answers 3, 4), 3 – Neutral (answers 5, 6), 4 – Complying most of the time (answers 7, 8), 5 – Complying completely (answers 9, 10) and presented as frequencies.

Variables related to the impact of Covid-19 on different areas of life were collected on a 1 to 10 scale (1 ‘No disruption at all, 10 ‘Serious disruption’) and the differences in answers between the two lockdowns were assessed using a two-sample t-test with unequal variances.

We used an alpha level of 0.05 for all statistical tests. All analyses were performed in Stata v.16. The graphs were created in Stata v.16 and Python.

## Results

### Participant characteristics

**Fig. 1:**
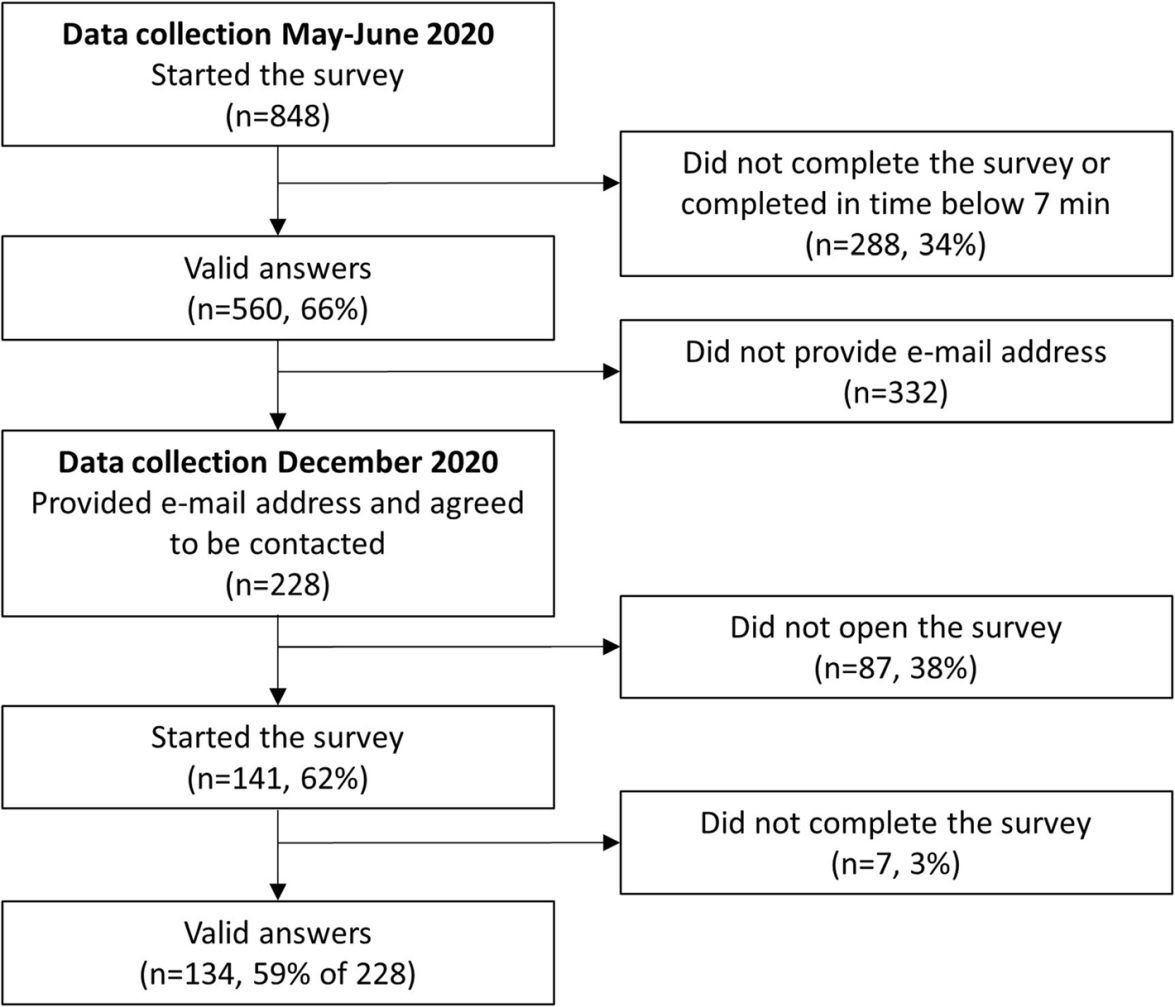
Recruitment of study participants

In the first wave of the survey (data collection in May–June 2020) valid answers were obtained from 560 participants. Of these 560 participants, 228 provided their e-mail addresses and agreed to be contacted in the future. From the 228 persons who were re-contacted during the second wave of the survey (data collection in December 2020), 141 responded among which 134 (59% response rate) provided valid answers and were included in the analysis of the second wave of the survey (Fig. 1).

Table 1 presents the characteristics of study participants during the two waves of data collection. Participants in the second wave were on average 3 years older. Otherwise, there was no significant difference in the main socio-demographic variables between the two surveys. Data relating to the socio-demographic characteristics of the sample compared to official Austrian population statistics, with respect to age, gender, distribution of population across federal states [35], migration background [36], education level [37], and employment status [38], are shown in Supplementary file 2 (Table 1A).

**Table 1:** Respondent characteristics in the two waves of data collection

	Lockdown March/April ***N*** = 560		Lockdown November/December ***N*** = 134		Difference
	N	%	N	%	
**Gender**					
Female	416	74%	98	73%	*χ*^*2*^(2, *N* = 694) = 1.24; *p* = .54
Male	143	26%	35	26%	
Diverse	1	0%	1	1%	
Missing	0	0%	0	0%	
**Age (Mean, SD)**	40.22	11.60	43.40	12.83	*t*(685) = −2.79; ***p*** **< .01**
18–29	97	17%	20	15%	χ^2^(3, *N* = 687) = 9.87**;** ***p*** **= .02**
30–49	319	57%	63	47%	
50–64	124	22%	45	34%	
65–79	13	2%	6	4%	
Missing	7	1%	0	0%	
**Federal state**					
Vienna	215	38%	62	46%	χ^2^(1, *N* = 694) = 2.80; *p* = .09
Other federal states	345	62%	72	54%	
Missing	0	0%	0	0%	
**Migration background**					
No migration background	489	87%	118	88%	χ^2^(1, *N* = 691) = 0.40; *p* = .53
Migration background	66	12%	13	10%	
Missing	5	1%	3	2%	
**Education**					
Primary education	13	2%	4	3%	χ^2^(2, *N* = 694) = 3.97; *p* = .14
Secondary education	245	44%	46	34%	
Higher education	302	54%	84	63%	
Missing	0	0%	0	0%	
**Employment status**					
Housekeeping	28	5%	5	4%	χ^2^(6, *N* = 694) = 3.49; *p* = .74
Student	37	7%	8	6%	
Employed	410	73%	95	70%	
Self-employed	37	7%	9	7%	
Unemployed	16	3%	4	3%	
Retired	25	4%	11	8%	
Missing	7	1%	2	2%	
**Family status**					
Single	204	36%	49	37%	χ^2^(4, *N* = 694) = 0.28; *p* = .99
Married or registered partnership	286	52%	69	51%	
Widowed	6	1%	1	1%	
Divorced	46	8%	10	8%	
Missing	18	3%	5	4%	
**Children**					
Yes	311	56%	77	57%	χ^2^(2, *N* = 694) = 1.07; *p* = .59
No	245	44%	57	43%	
Missing	4	1%	0	0%	
**Direct Covid-19 experience**	39	7%	8	6%	χ^2^(1, *N* = 694) = 0.17; *p* = .68
Tested positive for Covid-19	7	1%	3	2%	
Experienced symptoms of Covid-19, not tested	32	6%	5	4%	
Missing	0	0%	0	0%	
**Indirect Covid-19 experience**^**a**^	110	20%	73	58%	χ^2^(1, *N* = 694) = 67.58; ***p*** **< .01**
Close friend tested positive for Covid-19	46	9%	50	40%	
Family member tested positive for Covid-19	32	6%	32	25%	
Knew someone who died of Covid-19	44	8%	25	20%	
Missing	0	0%	0	0%	
**Quarantine or self-isolation in the past months**^**b**^					χ^2^(1, *N* = 694) = 8.61; ***p*** **< .01**
Yes	23	4%	14	10%	
No	537	96%	120	90%	
Missing	0	0%	0	0%	

Note: ^a^Respondents included in the “Direct Covid-19 experience” variable were excluded from this group;

^b^In the 1st Wave of data collection this question referred to the time from the beginning of the pandemic until time of data collection (May/June 2020); for the 2nd Wave of data collection, this question referred to the time since May 2020 until time of data collection (December 2020)

As indicated by the Covid-19-related variables, in the second wave of data collection significantly more respondents had indirect experience with Covid-19 through family or friends (*χ*^*2*^(1,*N* = 694) = 67.58, *p* < .001) compared to respondents in the first wave of the study. In the second wave of the study more respondents (10%) reported that they had to quarantine or self-isolate during the previous month due to Covid-19, compared to the first wave (4%) (χ^2^(1, *N* = 694) = 8.61; *p* < .01) (Table 1).

### Concern about Covid-19

**Fig. 2:**
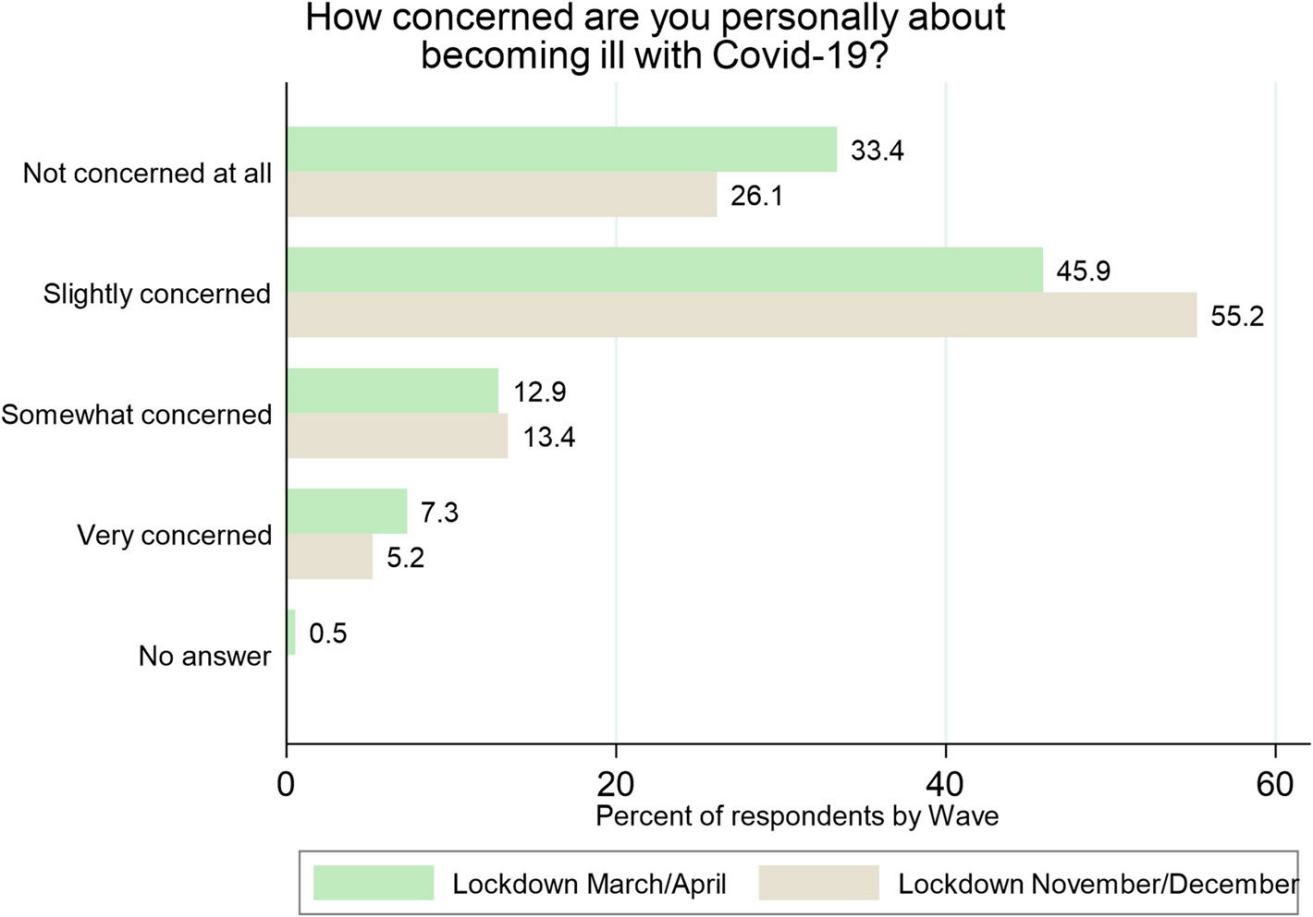
Concerned about Covid-19 illness

The greater spread of the coronavirus among the Austrian population during fall 2020 was well-indicated in the answers of the respondents about their concerns of contracting Covid-19. During the second lockdown in November/December, respondents were more concerned that they might become infected than during the first lockdown in March/April. In the first lockdown, 34% of participants were not concerned at all, leaving 66% of respondents somewhat, slightly or very concerned (Fig. 2). During the second lockdown, the proportion of study participants who were somewhat, slightly or very concerned about infection rose to 74%. However, the differences between the two lockdowns were not statistically significant (χ^2^(4, *N* = 694) = 5.19; *p* < .27). During the first lockdown, women were slightly more concerned than men, however, during the second lockdown this difference was no longer observable.

**Fig. 3:**
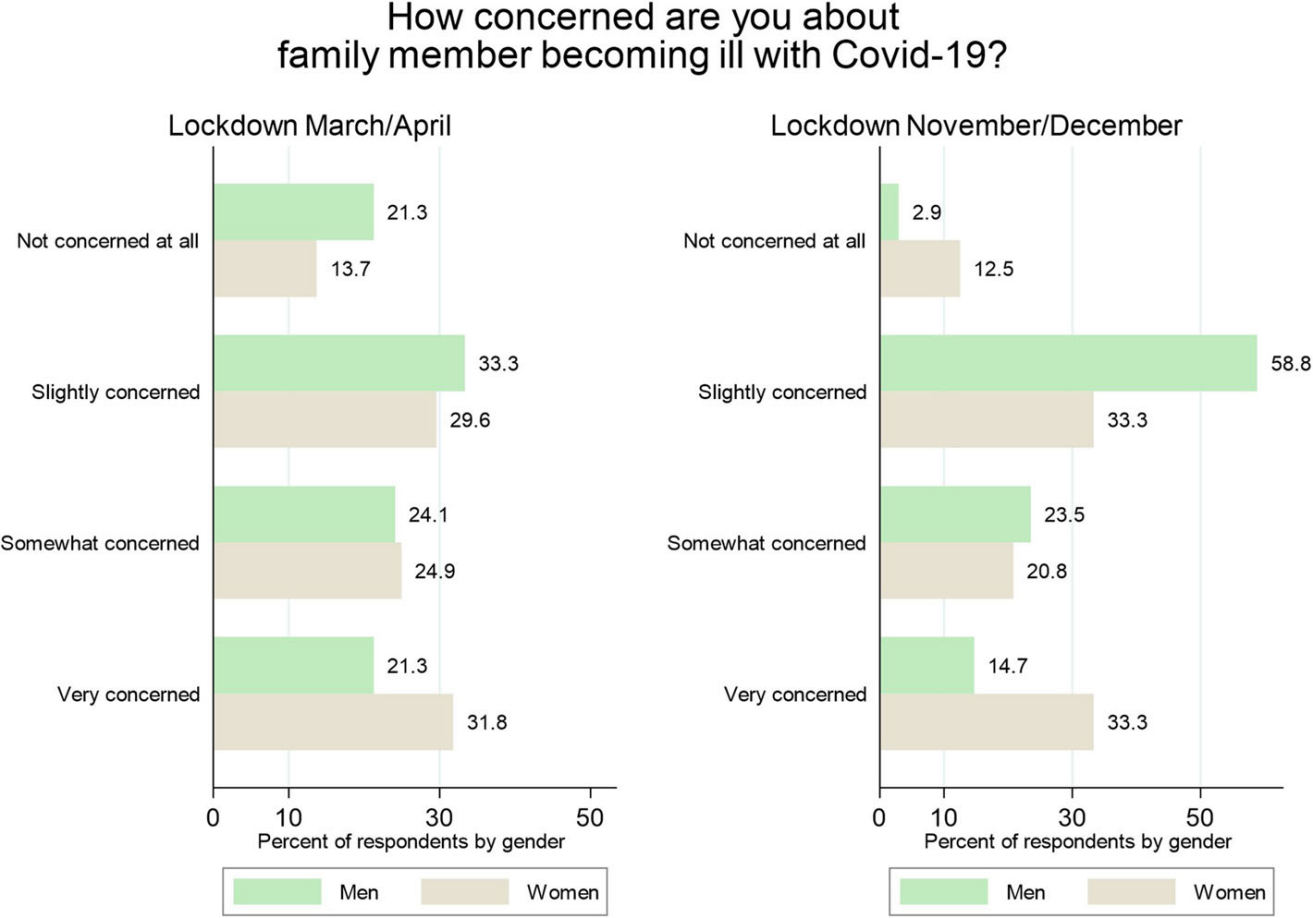
Concerned about family member becoming ill with Covid-19 by gender

Women were more concerned about their family members becoming infected than men. During the first lockdown, 32% of female respondents were ‘Very concerned’ compared to 21% of male respondents; during the second lockdown, 33% of females were ‘Very concerned’ as opposed to 15% of males. Change in the attitude to the risk of Covid-19 infection among men was observed. While during the first lockdown 21% of men reported having no concerns over their family members becoming infected with Covid-19, this proportion was reduced to just 3% during the second wave of the study (Fig. 3). Observed differences between men and women were statistically significant in the first lockdown (χ^2^(3, *N* = 550) = 8.35; *p* < .04) as well as in the second lockdown (χ^2^(3, *N* = 130) = 9.52; *p* < .02).

### Personal experience of the Covid-19 lockdowns

In the second wave of the study, substantially more respondents indicated that the Covid-19 lockdown did not pose any threat to their livelihood/income as compared to the first wave of data collection (59% in March/April and 84% in November/December) (χ^2^(4, *N* = 690) = 45.34; *p* < .01). However, this could be associated with the characteristics of study participants who, in comparison to the general population in Austria, had higher education level (Supplementary file 2 Table 1A). In the second lockdown, study participants felt slightly less isolated compared to the lockdown in March/April, however, not a statistically significant improvement (Table 2). Variables ‘I now know better what is really important in life’, ‘people being nicer because of the pandemic’ and ‘being more connected to the local community’ were significantly more pronounced in the first lockdown as compared to the second lockdown (Table 2). This finding suggests that unprecedented situation of the pandemic initially invoked strong feelings of solidarity, community, and appreciation which might have faded away with the prolonged time of the pandemic.

**Table 2:** Personal experience of the Covid-19 lockdowns

	Lockdown March/April		Lockdown November/December		Difference	Direction of difference	Test of difference^c^
Variable^a^	N	Mean (SD)	N	Mean (SD)			
Lockdown is a threat to my livelihood/income	556	2.43 (1.40)	134	1.58 (0.98)	−0.85	improved	χ^2^(4, *N* = 690) = 45.34; ***p*** **< .01**
It is more difficult than usual for me to focus on my work or my normal, daily activities	557	3.11 (1.36)	133	2.65 (1.39)	−0.46	improved	χ^2^(4, *N* = 690) = 14.19; ***p*** **< .01**
I am less busy than usual	558	2.34 (1.42)	134	2.19 (1.34)	−0.15	improved	χ^2^(4, *N* = 692) = 3.40; *p* = 0.49
I feel more isolated than usual	557	3.46 (1.36)	132	3.27 (1.33)	−0.18	improved	χ^2^(4, *N* = 689) = 4.17; *p* = 0.38
Variable^b^							
The lockdown restrictions are necessary	559	3.97 (1.21)	134	4.04 (1.21)	0.07	improved	χ^2^(4, *N* = 693) = 0.99; *p* = .91
I now know better what is really important in life	558	3.13 (1.19)	134	2.66 (1.12)	−0.46	worsened	χ^2^(4, *N* = 692) = 19.49; ***p*** **< .01**
I have been communicating with relatives more often	558	2.90 (1.15)	134	2.63 (1.06)	−0.28	worsened	χ^2^(4, *N* = 692) = 8.78; *p* = .07
I have a greater sense of appreciation for the healthcare workers	555	3.50 (1.18)	134	3.38 (1.06)	−0.11	worsened	χ^2^(4, *N* = 689) = 7.27; *p* = .12
People have become more friendly towards other people in my area	555	2.75 (1.06)	133	2.22 (0.85)	−0.53	worsened	χ^2^(4, *N* = 688) = 35.72; ***p*** **< .01**
I feel more connected to the members of my local community	559	2.57 (1.11)	133	2.06 (0.99)	−0.51	worsened	χ^2^(4, *N* = 692) = 25.23; ***p*** **< .01**

Note: ^a^Answers to the question “Indicate how much you agree/disagree with the following statement “were given on 1 to 5 Likert scale (1 – Strongly disagree, 2 – Slightly disagree, 3 – Neutral, 4 – Slightly agree, 5 – Strongly agree), lower score represents better outcome; ^b^Higher score represents better outcome; ^c^Pearson’s chi-square test

### Perceptions of imposed public health measures

During both lockdowns, about three-quarters of the study respondents agreed that the implemented public health measures restricting movement of members of the general public during the Covid-19 pandemic, were necessary to limit the outbreak of the virus (74% in March/April and 77% in November/December) (Fig. 1A in Supplementary file 2).

With respect to the accessibility of sufficient information surrounding the pandemic, during the first lockdown in March/April, respondents were more convinced that they received clear advice about the Covid-19 situation from the government, compared to the second lockdown in November/December (χ^2^(3, *N* = 694) = 15.81; *p* **<** .01) (Fig. 2A in Supplementary file 2). In March/April two-thirds (66%) of all respondents felt that the government provided sufficient information about the Covid-19 situation (31% did not agree with the statement, and 3% provided no answer), while in November/December less than a half (48%) agreed with this statement (48% did not agree, and 4% provided no answer). There were no statistically significant differences between male and female participants in either wave of the study. Interestingly, during both lockdowns, participants with migration background reported they felt they received more clear advice from government, compared to those without migration background (73% vs. 65% during the first lockdown, 61% vs. 46% during the second lockdown) (Fig. 3A in Supplementary file 2).

Perceptions of the necessity of certain measures changed between the first and the second lockdown with respect to wearing masks in public closed spaces which gained substantial support over time. While wearing masks was rated as the most unnecessary measure during the first lockdown, it was rated as the most essential one during the second lockdown; 41% of respondents seeing this measure as absolutely essential in March/April, compared to 80% in November/December (Table 3). The lowest acceptance among study participants during the second lockdown was associated with closure of schools and distance learning with only 22% seeing this as an absolutely essential measure. During the first lockdown, regulations about physical contact with family from different household was rated the least essential.

**Table 3:** Perceptions of the necessity of public health measures

On a scale of 1 to 10 (ranging from 1 ‘Completely unnecessary’ to 10 ‘Absolutely essential’) please indicate how necessary you think the following lockdown restrictions were to contain the Covid-19?					
**Lockdown March/April**			**Lockdown November/December**		
	Completely unnecessary^a^	Absolutely essential^a^		Completely unnecessary^a^	Absolutely essential^a^
Commuting to and from work only when absolutely necessary	**8%**	**48%**	Restrictions on leaving private living space	16%	31%
Walks only with people living in the same household	15%	32%	Distance of one meter in public space for people from different households	6%	71%
Closure of all non-essential shops and business premises	15%	37%	Closure of all non-essential shops and business premises	13%	38%
Only necessary purchases e.g. groceries, medication	11%	47%	School closings and distance learning	*26%*	*22%*
No physical contact with family members outside the same household	*20%*	*27%*	Physical contact only with closest relatives or individual important caregivers	14%	40%
Mouth and nose protection in open business premises and public transport	*20%*	41%	Mouth and nose protection in open business premises and on public transport	**5%**	**80%**
			Visits in nursing homes and hospitals once a week	12%	34%
			Switch to home office wherever possible	**5%**	63%

Note: The most essential measures as indicated by respondents are in **bold**, the least essential are in *italics*.

^a^Answers related to the necessity of the introduced public health measures reported by the study participants on the 1-10 scale were re-coded to a 5-point scale: 1 – Completely unnecessary (answers 1, 2), 2 – Unnecessary (answers 3, 4), 3 – Neutral (answers 5, 6), 4 – Necessary (answers 7, 8), 5 – Absolutely essential (answers 9, 10)

Fig. 4A and Fig. 5A in Supplementary file 2 reflect age, gender and regional heterogeneity of respondents’ opinions during the two lockdowns (Fig. 4A in March/April, Fig. 5A in November/December). While the support for different measures during the first lockdown was similarly distributed among the age groups, there seemed to be more support from younger age groups (18–39 and 30–49) for measures forbidding physical contact with family members outside the same household and closure of non-essential businesses. Women expressed more approval for most of the measures compared to men, as well as people living in Vienna compared to respondents from the remaining federal states. During the second lockdown in November/December, closure of schools and distance learning was distinctly less supported by females, compared to males.

### Compliance with public health measures

In terms of complying with the imposed public health measures during both lockdowns, respondents complied the least with restrictions limiting contact with relatives outside the same household (Table 4). While 63% fully complied with this measure during the first lockdown, only half of the participants (50%) did the same during the second lockdown. At both points of data collection respondents reported complying the most with wearing a mask in indoor public spaces (91 and 95% in the first and second lockdown, respectively). Reduced compliance with restrictions on leaving private living spaces was observed during the second lockdown, with only 58% of study respondents reporting full compliance with this measure as opposed to 83% who reported complying completely with commuting to and from work only when absolutely necessary in March/April.

**Table 4:** Compliance with the public health measures during lockdowns

On a scale of 1 to 10 (ranging from 1 ‘Not complying at all’ to 10 ‘Complying completely’) please indicate how much were you complying with the following lockdown restrictions?					
**Lockdown March/April**			**Lockdown November/December**		
	Not complying at all^a^	Complying completely^a^		Not complying at all^a^	Complying completely^a^
Commuting to and from work only when absolutely necessary	5%	83%	Restrictions on leaving private living space	8%	58%
Walks only with people living in the same household	5%	73%	Distance of one meter in public space for people from different households	3%	71%
Only necessary purchases e.g. groceries, medication	4%	81%	Switch to home office wherever possible	*13%*	67%
No physical contact with family members outside the same household	*6%*	*63%*	Physical contact only with closest relatives or individual important caregivers	10%	*50%*
Mouth and nose protection in open business premises and public transport	**2%**	**91%**	Mouth and nose protection in open business premises and on public transport	**1%**	**95%**

Note: Measures with which respondents complied the most are in **bold**, the least – in *italics*.

^a^Answers related to the complying with the lockdown restrictions reported by the study participants on the 1-10 scale were re-coded to a 5-point scale: 1 – Not complying at all (answers 1, 2), 2 – Not always complying (answers 3, 4), 3 – Neutral (answers 5, 6), 4 – Complying most of the time (answers 7, 8), 5 – Complying completely (answers 9, 10)

With respect to differences in compliance by age, gender and federal state, women and respondents from Vienna reported an overall higher level of adherence to all public health measures during both lockdowns, as compared to men and respondents from other federal states, respectively. The youngest age group (18–29) reported slightly lower compliance with meeting only people from the same household in the first lockdown and with physical distancing during the second lockdown, compared to older age groups (Fig. 6A and Fig. 7A in Supplementary file 2).

### The impact of Covid-19 on various aspects of life

On average, respondents reported more disruptions in all areas of their lives during the second lockdown in November/December compared to the first lockdown in March/April (Table 5). In both waves of the study, the most impact of the Covid-19 pandemic was reported in association with leisure activities (mean of 6.4 on 1 to 10 scale in March/April, and 7.5 in November/December, the higher score represents more disruptions) and community life (mean of 6.2 in March/April and 7.3 in November/December), while the lowest impact was given to spirituality. The impacts on friendships, family life, education, leisure and community life were significantly more disrupted during the second wave of the survey.

**Table 5:** Impact of the Covid-19 pandemic on various aspects of life

On a scale of 1 to 10 indicate how much the Covid-19 has impacted on the following domains of your life (1 ‘No disruption at all, 10 ‘Serious disruption’)								
**Lockdown March/April**				**Lockdown November/December**				
	Mean (SD)	1 ‘No disruption at all’	10 ‘Serious disruption’		Mean (SD)	1 ‘No disruption at all’	10 ‘Serious disruption’	Change in mean^a^
Impact on family life	3.80 (2.90)	36%	5%	Impact on family life	4.70 (2.68)	19%	5%	0.90**
Impact on marriage	3.39 (2.99)	45%	7%	Impact on marriage	3.56 (2.94)	36%	8%	0.17
Impact on parenting	2.93 (2.66)	56%	**2%**	Impact on parenting	3.45 (2.86)	44%	**3%**	0.52
Impact on friendships	5.28 (3.00)	18%	11%	Impact on friendships	6.73 (2.45)	5%	12%	1.45***
Impact on work	4.60 (3.22)	28%	11%	Impact on work	5.09 (3.06)	21%	11%	0.49
Impact on education	3.83 (3.29)	47%	9%	Impact on education	4.94 (3.32)	28%	11%	1.11**
Impact on leisure	*6.43 (3.01)*	*13%*	*21%*	Impact on leisure	*7.51 (2.54)*	*6%*	*30%*	1.08***
Impact on spirituality	**2.84 (2.72)**	**59%**	5%	Impact on spirituality	**3.19 (2.82)**	**53%**	5%	0.35
Impact on community	6.24 (3.08)	14%	20%	Impact on community	7.27 (2.76)	9%	26%	1.03***
Impact on physical self-care (diet, exercise, sleep)	3.38 (2.81)	44%	3%	Impact on physical self-care (diet, exercise, sleep)	3.85 (2.76)	28%	6%	0.47

Note: The areas impacted the least are in **bold**, the areas impacted the most are in *italics*.

^a^Two-sample t test with unequal variances; *p<.05, **p<.01, ***p<.001

## Discussion

The study offers a comprehensive insight into experiences of the Covid-19 pandemic situation as well as perceptions and attitudes towards imposed public health measures of the general population during the two lockdowns in Austria. More specifically, the study provides a snapshot of people’s opinions, concerns, personal experiences and perceptions of and compliance with preventive measures throughout the Covid-19 pandemic in Austria during the two lockdowns in March/April and November/December 2020. Based on the collected data, we can draw conclusions from the two confinement stages in the Covid-19 pandemic that can facilitate the discussion around the design of future lockdown strategies and confinement policies.

Our findings suggest that, compared to the first lockdown in March/April, the time period during the second lockdown in November/December appeared to have significantly more negative effects in terms of personal experiences of attachment to the local community, appreciation of healthcare workers and people around, and feeling of understanding better what really matters in life. It also caused more disruption to friendships, leisure activities, and community and family lives. Furthermore, compliance with the government-imposed restrictions reduced between the two lockdowns, except for the use of masks and face coverings in indoor public spaces. For instance, we observed the reduction in compliance with restrictions on leaving private dwellings during the second lockdown. This result confirms the analysis of the mobile phone mobility data during the two lockdowns in Austria which showed that there was a clear change from lockdown to lockdown. While the observed reduction in mobility across nine federal states varied between − 57% and − 80% during the first lockdown in March/April, the reduction observed during the lockdown in November/December was lower and ranged from − 30% to − 50%, as compared to the weeks preceding the introduction of restrictions [39]. Moreover, the perceived necessity and compliance with some of the public health measures was different among age groups, gender and regions of Austria. For instance, our study found that the compliance with the Covid-19 lockdown measures was lower among men compared to women. These results are consistent with similar international studies [35, 36].

The findings on the high support with the mandatory face mask use in public spaces observed in our study is in line with the findings of the Austrian Corona Panel Project which also showed the highest support for this public health measure. In our study, 80% of study participants saw this measure as absolutely essential during the second lockdown, compared to 72% participants of the Austrian Corona Panel Project during a comparable time period [30]. The compliance to this measure was the highest of all presented measures in our study during both lockdowns which can be explained by an increased police presence and fines for not wearing a mask.

In the second wave of the study, more respondents indicated that the Covid-19 lockdown did not pose any threat to their livelihood/income as compared to the first wave of data collection. This could be associated with the characteristics of study participants who, in comparison to the general population in Austria, were more educated (54% in the study sample in the first wave of data collection and 63% in the second wave of data collection, compared to 13% in the general population [37]). A recent study from Austria showed that the negative effects of the Covid-19 pandemic were most notable in lower socio-economic groups especially in the case of job loss, decrease in financial stability, and declining mental health [40]. A smaller proportion of respondents felt more isolated since the beginning of the second lockdown in November/December, when compared to the first lockdown. This may be related to the slightly different regulations during the second lockdown in Austria which allowed for visitation of close relatives and important caregivers as well as partners who do not live in the same household which was not officially allowed during the first lockdown.

Two-thirds (66%) of respondents in our study agreed that the government provided sufficient information about the Covid-19 pandemic situation in March/April, compared to nearly 68% of respondents of the Austrian Corona Panel Project who reported in March that they were very or somewhat satisfied with the work of the government. When compared to other countries, during a similar time period, 45 and 61% of study respondents in Norway and Sweden, respectively, strongly agreed or agreed that they trusted their government during the first wave of the pandemic in March and April 2020 [41]. In our study the proportion of respondents who agreed that the government provided sufficient Covid-19 advice decreased by 18% between the first and the second lockdown. At the same time, the Austrian Corona Panel Project reported almost 50% decrease in satisfaction with the government’s work between March and December 2020 [42]. Together these findings suggest a negative trend over time in relation to the perceived effectiveness and transparency of the government actions taken during the pandemic.

Our study showed that during the first lockdown people rated their experiences related to the attachment to the local community, appreciation of life and healthcare workers, and people around being more friendly higher, compared to 7 months later during the second lockdown. Similarly, in the Austrian Corona Panel Project, the initially very positive assessment of the development of social cohesion continued to decline with each wave of surveys since March 2020 [43]. It is not clear whether this decline is related to the general decrease in the perceived social cohesion and solidarity, or the fact that the prolongation of the pandemic and recurring lockdowns made people lose hope and confidence that the situation would resolve and holding together as a community could end this crisis sooner.

Since the Covid-19 pandemic is far from being over and future lockdowns are inevitable, there are a few lessons we can learn from this study that may be useful in future planning of confinement policies. Firstly, our findings show that the compliance with certain restrictions decreased between the first and the second lockdown which raises a question about the effectiveness of future lockdown measures due to declining compliance. A study from the United States also found that as the pandemic progressed, both younger and older people tended to resume potentially risky social behaviours, especially in terms of visiting friends and family [44]. This aspect should be considered when designing new measures as well as the timing and length of future lockdowns in addition to certain support measures (e.g. availability of free testing, psychological and financial support services) that could improve compliance and lessen social and economic disruption. Secondly, according to our data, closure of schools had a very low approval among study participants, especially among women. This indicates an increased burden related to childcare and home-schooling during the pandemic experienced by women. This should be taken into account when planning further school and childcare facility closures. As outlined by Power (2020), specific policies during the Covid-19 pandemic should focus on support and protection for unpaid care-givers, including subsidies to replace pay for workers who are unable to work due to the closure of schools and daycare facilities, expanding access to paid family leave and paid sick leave, and extending benefits to those resigning from employment to provide child care due to the pandemic [[Bibr CR45]]. Thirdly, we observed that people felt that they did not receive sufficient information about the Covid-19 situation from the government during the second lockdown, when compared to the first lockdown. This is concerning as this finding might also indicate that also the trust in the information received from the government decreased as the pandemic progressed. Studies indicate associations between the political trust and social distancing practices by members of the general population [46] and overall compliance with restrictions during the Covid-19 pandemic [47]. Trust in official governmental media proved to be an independent predictor of protective behaviours in a study from China [48]. Since adherence to government-imposed restrictions by the general population is key in containing Covid-19, the focus should be on restoring political trust of the general population.

Some limitations of this study need to be considered. The study sample was collected via online adverts and the responses may not be generalizable to the whole Austrian population. The majority of the participants were women, and there was an overrepresentation of the age group 30 to 49 years and underrepresentation of the age group above 65 years [35] as well as overrepresentation of people with higher education [37], compared to the general population. This is a common limitation observed in online surveys. Previous research has shown that younger age [49, 50] and higher education [50, 51] predict higher willingness to participate in online surveys. Furthermore, the presented analysis is mostly descriptive and any tests of differences are conducted on unadjusted data. However, since the survey in the second wave of the study was conducted on a sub-group of participants from the first wave, and there were no significant differences between the two groups in terms of participant characteristics, the presented results outlining differences observed between the two lockdowns are robust for this sample.

## Supplementary Information

**Additional file 1.**

**Additional file 2.**

## Data Availability

The datasets generated during the current study and the study protocol have been released in a scientific data repository and can be accessed through the link: https://zenodo.org/record/4598821.
