# Supplementary material for: Perceptions of Covid-19 lockdowns and related public health measures in Austria: a longitudinal online survey
Source: BMC Public Health. 2021 Aug 4;21:1502. doi: 10.1186/s12889-021-11476-3 (PMC8331215; doi:10.1186/s12889-021-11476-3)
Supplement: Supplementary file 1 — Additional file 1. [file 12889_2021_11476_MOESM1_ESM.docx]

**Supplementary file 1**

**Perceptions of Covid-19 lockdowns and related public health measures in Austria: a longitudinal online survey**

Agata Łaszewska^1^, Timea Helter^1^, Judit Simon^1^

^1^ Medical University of Vienna, Center for Public Health, Department of Health Economics, Kinderspitalgasse 15, 1090 Vienna, Austria

**Corresponding author:** Agata Łaszewska, Medical University of Vienna, Center for Public Health, Department of Health Economics, Kinderspitalgasse 15, 1090 Vienna, Austria, Phone: +43 1 40160-34844, E-Mail: [agata.laszewska@meduniwien.ac.at](mailto:agata.laszewska@meduniwien.ac.at)

**Journal:** BMC Public Health

**List of questionnaire sections:**

1. Demographic section
2. Covid-19-related questions
3. Lockdown-related questions
4. **Demographic section**
5. Gender

Male

Female

Diverse

1. What is your age? (Years from 18 to 90 in numbers). If you prefer not to give this information then please leave it blank.
2. Do you have a migration background?

No

EU countries prior to 2004 / EEA / Switzerland

EU accession countries from 2004

Former Yugoslavia (non-EU), Turkey

Other countries

1. What is your highest educational achievement?

Primary school

Apprenticeship with vocational school

Technical or commercial school

“Matura”

Degree from a university, (technical) college

Any other higher degree following “Matura”

1. In which federal state was your main residence?

Burgenland

Carinthia

Lower Austria

Upper Austria

Salzburg

Styria

Tyrol

Vorarlberg

Vienna

1. What is your marital status?

Single

Married or registered partnership, living together

Married or registered partnership, separated

Widowed

Divorced

I prefer not to provide this information

1. Do you have any children?

Yes

No

I prefer not to provide this information

1. If yes, how many?
2. What is your employment status?

I was not employed (e.g. household)

Student

Employed

Self-employed

Civil servant

Unemployed

Retired

I prefer not to provide this information

1. In the past 7 months (since mid-April) have you been fired due to COVID-19?

Yes

No

I prefer not to provide this information

1. In the past 7 months (since mid-April) have you been sent to short-term working (“Kurzarbeit”) due to COVID-19?

Yes

No

I prefer not to provide this information

1. Are you currently receiving treatment for (yes/no/prefer not to say):

Diabetes

Heart/cardiovascular disease

Stroke/cerebrovascular disease

Lung disease (e.g. Asthma, Cystic Fibrosis, COPD)

Liver disease (e.g. Hepatitis)

Cancer

1. **COVID-19-related questions**
2. Since mid-April I have tested positive for COVID-19.

Yes

No

1. Since mid-April I have experienced the symptoms of COVID-19 including persistent cough and high body temperature (above 37.8 degrees Celsius) since the outbreak in Austria began but was not tested.

Yes

No

1. I have a close friend who has tested positive for COVID-19 since mid-April.

Yes

No

1. I have one or more immediate family members who have tested positive for COVID-19 since mid-April.

Yes

No

1. I have known someone who died as a consequence of COVID-19 since mid-April.

Yes

No

1. In the past 7 months (since mid-April) I have had to self-quarantine because I have tested positive or had symptoms of COVID-19 or for any other reason.

Yes

No

1. **Lockdown-related questions**
2. How concerned are you personally about becoming infected with COVID-19?

Not concerned at all

Slightly concerned

Somewhat concerned

Very concerned

Prefer not to say

1. How concerned are you personally about a member of your family becoming infected with the COVID-19 virus?

Not concerned at all

Slightly concerned

Somewhat concerned

Very concerned

Prefer not to say

1. Do you feel that you have been provided with enough clear advice about COVID-19 from the government?

Yes

No

Prefer not to say

1. Have you found it difficult to switch off from media (TV news, newspapers) reports about COVID-19?

Yes

No

Prefer not to say

1. Have you found it difficult to switch off from social media (Facebook, Twitter etc) reports about COVID-19?

Yes

No

Prefer not to say

1. What is your preferred method of assessing information and updates regarding COVID-19? (Only one answer)

TV News outlets

Print newspapers

News websites

Online government platform

Social media

International guidelines (e.g. WHO)

Other

Prefer not to say

1. Indicate how much you agree/disagree with the following statement (Strongly disagree, Slightly disagree, Neutral, Slightly agree, Strongly agree

“The second lockdown in November 2020 is a threat to my livelihood/income.”

“Because of the threat of COVID-19, it is more difficult than usual for me to concentrate on my work or my normal, daily activities.”

“The measures restricting movement in public spaces are needed to limit the second spread of COVID-19.”

“Since the beginning of the second lockdown in November 2020, I have been less busy than usual.”

“Since the beginning of the second lockdown in November 2020, I now know better what really matters in life.”

“Since the beginning of the second lockdown in November 2020, I have been communicating with relatives more often.”

“Since the beginning of the second lockdown in November 2020, I have had a greater sense of appreciation and I am proud of the healthcare staff.”

“Since the beginning of the second lockdown in November 2020, I have felt more isolated than usual.”

“Since the beginning of the second lockdown in November 2020, I have the feeling that people have become more friendly towards other people in my area.”

“Since the beginning of the second lockdown in November 2020, I have felt more connected to members of my local community.”

1. On a scale of 1 to 10 (ranging from 1 ‘Completely unnecessary’ to 10 ‘Absolutely essential’) please indicate how necessary you think the following lockdown restrictions were to contain the Covid-19?

Restrictions on leaving private living space

Distance of one meter in public space for people from different households

Closure of all non-essential shops and business premises

School closings and distance learning

Physical contact only with closest relatives or individual important caregivers

Mouth and nose protection in open business premises and on public transport

Visits in nursing homes and hospitals once a week

Ban on events or restrictions in the event area

Switch to home office wherever possible

1. On a scale of 1 to 10 (ranging from 1 ‘Not complying at all’ to 10 ‘Complying completely’) please indicate how much were you complying with the following lockdown restrictions?

Restrictions on leaving private living space

Distance of one meter in public space for people from different households

Switch to home office wherever possible

Physical contact only with closest relatives or individual important caregivers

Mouth and nose protection in open business premises and on public transport

1. On a scale of 1 to 10 indicate how much the Covid-19 has impacted on the following domains of your life (1 ‘No disruption at all, 10 ‘Serious disruption’)

Family (other than marriage or parenting)

Marriage/couples/intimate relations

Parenting

Friends/social life

Work

Education/training

Recreation/fun

Spirituality

Community Life

Physical self-care (diet, exercise, sleep)
