# Supplementary material for: Perceptions of Covid-19 lockdowns and related public health measures in Austria: a longitudinal online survey
Source: BMC Public Health. 2021 Aug 4;21:1502. doi: 10.1186/s12889-021-11476-3 (PMC8331215; doi:10.1186/s12889-021-11476-3)
Supplement: Supplementary file 2 — Additional file 2. [file 12889_2021_11476_MOESM2_ESM.docx]

**Supplementary file 2**

**Perceptions of Covid-19 lockdowns and related public health measures in Austria: a longitudinal online survey**

Agata Łaszewska^1^, Timea Helter^1^, Judit Simon^1^

^1^ Medical University of Vienna, Center for Public Health, Department of Health Economics, Kinderspitalgasse 15, 1090 Vienna, Austria

**Corresponding author:** Agata Łaszewska, Medical University of Vienna, Center for Public Health, Department of Health Economics, Kinderspitalgasse 15, 1090 Vienna, Austria, Phone: +43 1 40160-34844, E-Mail: [agata.laszewska@meduniwien.ac.at](mailto:agata.laszewska@meduniwien.ac.at)

**Journal:** BMC Public Health

**Table 1A**. Study sample characteristics in comparison to the general population in Austria

|  | **Lockdown March/April**  **N=560** | | **Lockdown November/December**  **N=134** | | **Austrian population** |
| --- | --- | --- | --- | --- | --- |
|  | N | % | N | % | % |
| **Gender** |  |  |  |  |  |
| Female | 416 | 74% | 98 | 73% | 51% |
| Male | 143 | 26% | 35 | 26% | 49% |
| Diverse | 1 | 0% | 1 | 1% |  |
| Missing | 0 | 0% | 0 | 0% |  |
| **Age (Mean, SD)** | 40.22 | 11.60 | 43.40 | 12.83 |  |
| 18-29 | 97 | 17% | 20 | 15% | 18% |
| 30-49 | 319 | 57% | 63 | 47% | 34% |
| 50-64 | 124 | 22% | 45 | 34% | 26% |
| 65-79 | 13 | 2% | 6 | 4% | 23% |
| Missing | 7 | 1% | 0 | 0% |  |
| **Federal state** |  |  |  |  |  |
| Vienna | 215 | 38% | 62 | 46% | 21% |
| Other federal states | 345 | 62% | 72 | 54% | 79% |
| Missing | 0 | 0% | 0 | 0% |  |
| **Migration background** |  |  |  |  |  |
| No migration background | 489 | 87% | 118 | 88% | 84% |
| Migration background | 66 | 12% | 13 | 10% | 16% |
| Missing | 5 | 1% | 3 | 2% |  |
| **Education** |  |  |  |  |  |
| Primary education | 13 | 2% | 4 | 3% | 26% |
| Secondary education | 245 | 44% | 46 | 34% | 61% |
| Higher education | 302 | 54% | 84 | 63% | 13% |
| Missing | 0 | 0% | 0 | 0% |  |
| **Employment status** |  |  |  |  |  |
| Housekeeping | 28 | 5% | 5 | 4% | 2% |
| Student | 37 | 7% | 8 | 6% | 4% |
| Employed | 410 | 73% | 95 | 70% | 64% |
| Self-employed | 37 | 7% | 9 | 7% | 9% |
| Unemployed | 16 | 3% | 4 | 3% | 2% |
| Retired | 25 | 4% | 11 | 8% | 19% |
| Missing | 7 | 1% | 2 | 2% |  |

**Fig 1A.** Lockdown measures are necessary to limit spread of Covid-19

**Fig 2A.** Clear advice from the government

**Fig 3A.** Clear advice from the government by migration background


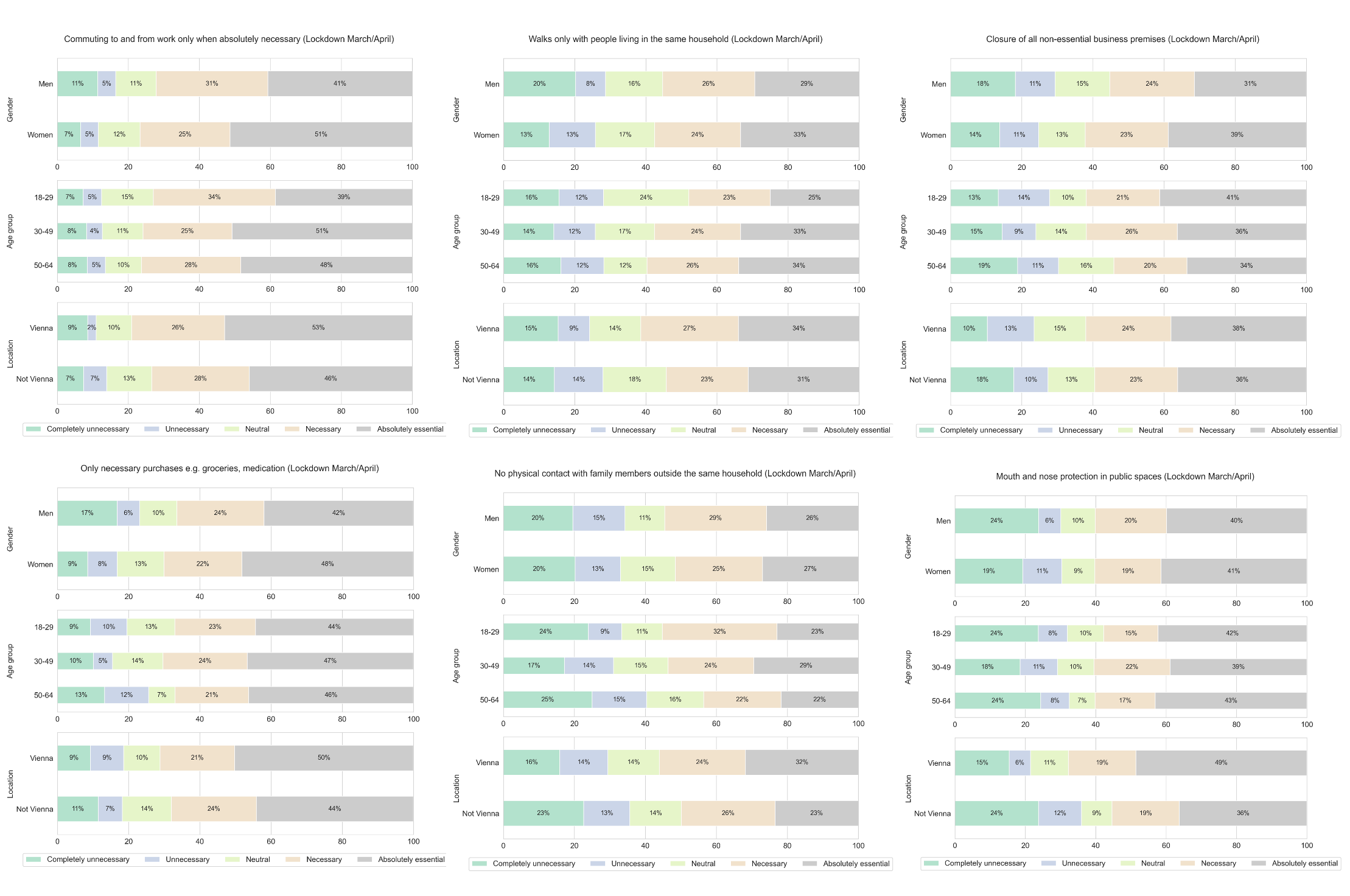


**Fig 4A.** Perceptions of the necessity of the public health measures during the first lockdown in March/April in the view of respondents by age, gender and federal state


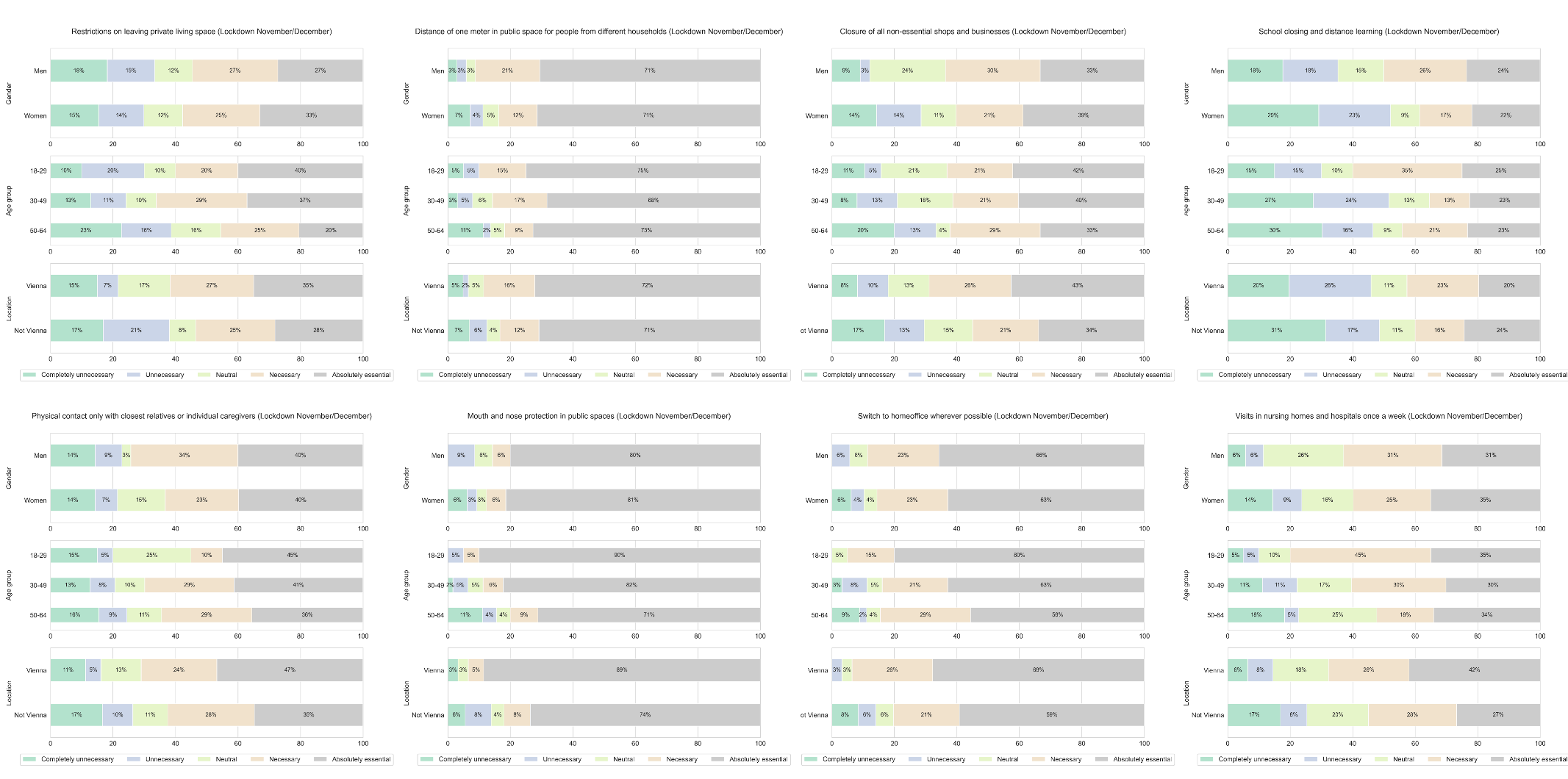


**Fig 5A.** Perceptions of the necessity of the public health measures during the second lockdown in November/December in the view of respondents by age, gender and federal state


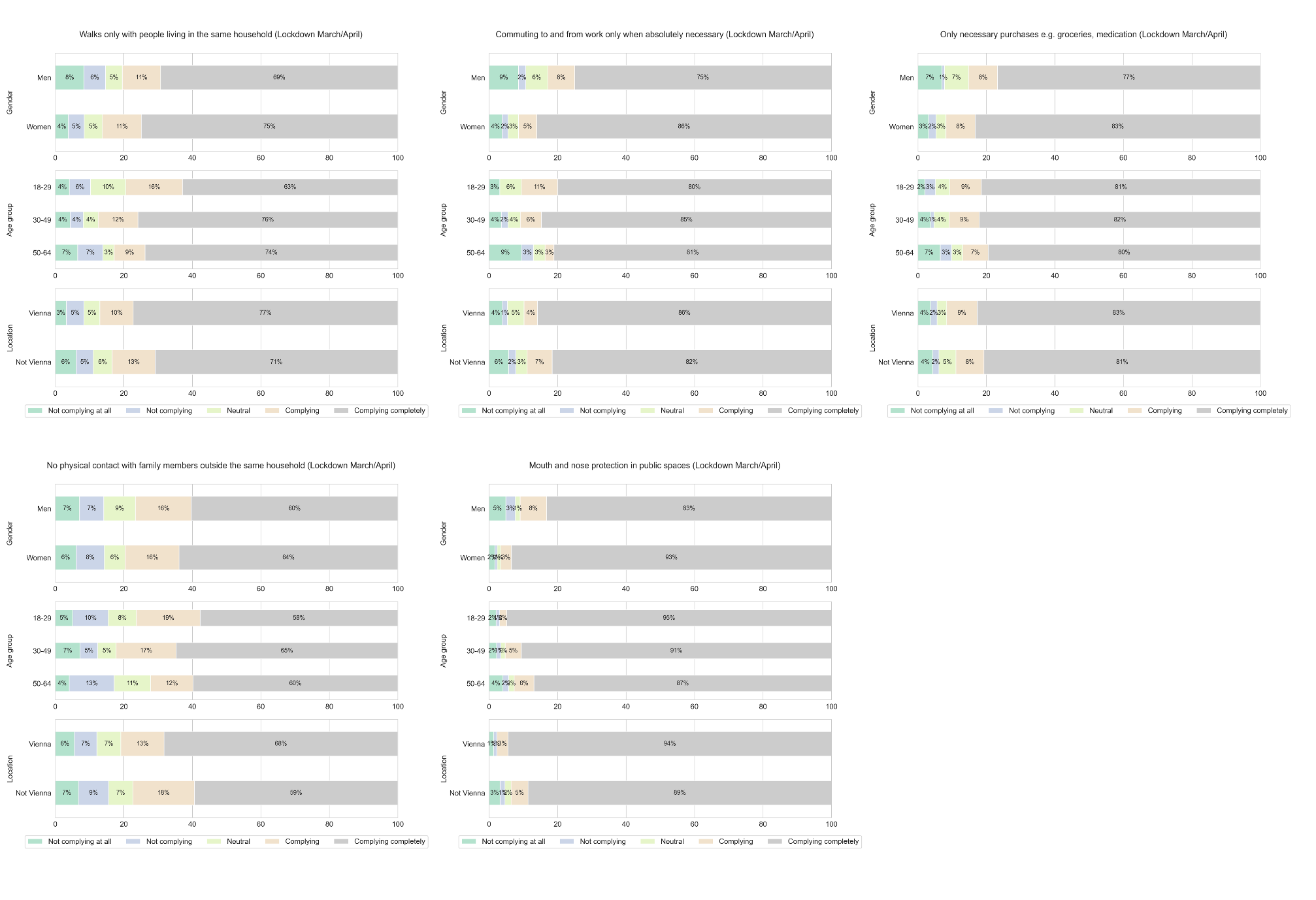


**Fig 6A.** Complying with the public health measures during the first lockdown in March/April in the view of respondents by age, gender and federal state


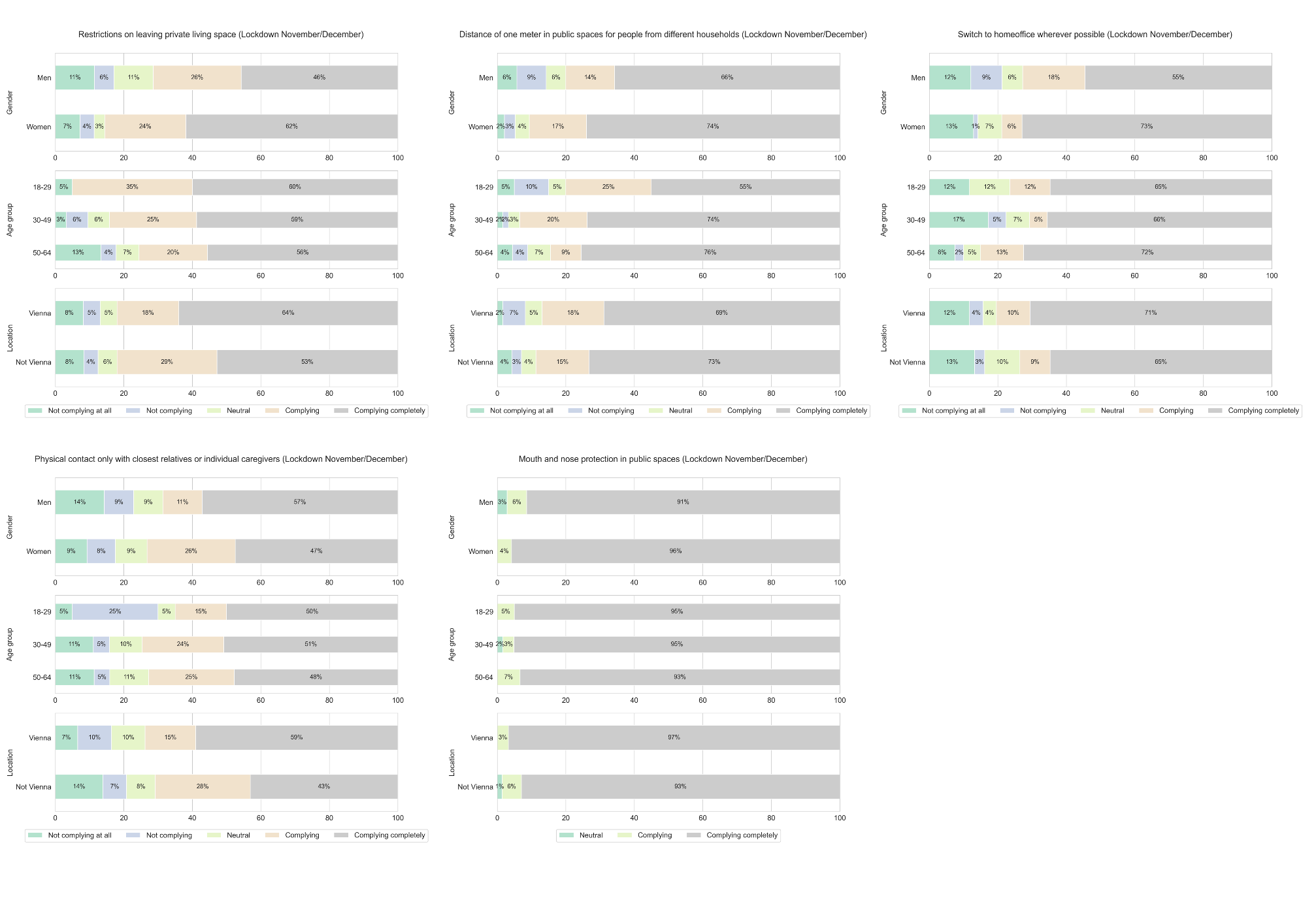


**Fig 7A.** Complying with the public health measures during the second lockdown in November/December in the view of respondents by age, gender and federal state
